# Risk of Venous Thromboembolism in Patients Nursed at Home or in Long-Term Care Residential Facilities

**DOI:** 10.1155/2011/305027

**Published:** 2011-06-13

**Authors:** Guido Arpaia, Federico Ambrogi, Maristella Penza, Aladar Bruno Ianes, Alessandra Serras, Patrizia Boracchi, Claudio Cimminiello

**Affiliations:** ^1^Departimento di Medicina, Azienda Ospedaliera di Desio e Vimercate, Via SS Cosma e Damiano 10, 20059 Vimercate, Italy; ^2^Departimento di Medicina del Lavoro “Clinica del lavoro Luigi Devoto”, Sezione di Statistica Medica e Biometria “G.A. Maccacaro”, Università degli Studi di Milano, 20133 Milan, Italy; ^3^Distretto Socio-Sanitario of Vimercate, ASL Monza and Brianza, 20900 Monza, Italy; ^4^Medical Area Gruppo Segesta, Viale Cassala 16, 20143 Milan, Italy; ^5^Unità di Chirurgia Vascolare, Policlinico di Monza, 20129 Milano, Italy

## Abstract

*Background*. This study investigated the prevalence of and impact of risk factors for deep venous thrombosis (DVT) in patients with chronic diseases, bedridden or with greatly limited mobility, cared for at home or in long-term residential facilities. *Methods*. We enrolled 221 chronically ill patients, all over 18 years old, markedly or totally immobile, at home or in long-term care facilities. They were screened at the bedside by simplified compression ultrasound. *Results*. The prevalence of asymptomatic proximal DVT was 18% (95% CI 13–24%); there were no cases of symptomatic DVT or pulmonary embolism. The best model with at most four risk factors included: previous VTE, time of onset of reduced mobility, long-term residential care as opposed to home care and causes of reduced mobility. The risk of DVT for patients with reduced mobility due to cognitive impairment was about half that of patients with cognitive impairment/dementia. *Conclusions*. This is a first estimate of the prevalence of DVT among bedridden or low-mobility patients. Some of the risk factors that came to light, such as home care as opposed to long-term residential care and cognitive deficit as causes of reduced mobility, are not among those usually observed in acutely ill patients.

## 1. Introduction

Quantifying the risk for patients with a risk of venous thromboembolism (VTE) persisting for a long time is still a problem as regards prophylaxis. These are typically chronic cases with reduced mobility who are cared for at home and outpatients—often elderly—entering long-term residential nursing facilities for chronic conditions. Although it has been demonstrated that nursing home residency is a risk factor for VTE [1] and these that patients are sometimes considered at permanent risk [2], it seems clear that not all will need pharmacological prophylaxis [3]. Indeed, no studies have been done yet to assess the benefit of prophylaxis and the most appropriate duration for these conditions. We do not even know the level of risk for VTE in these patients or the real risk factors. Advanced age and limited mobility alone are insufficient reasons for prescribing prophylaxis [4] but it is important to identify among elderly chronic patients with reduced mobility those with additional factors that raise their risk profile for VTE.

Investigating these populations is complicated, however, by logistic difficulties for instrumental screening for bedridden patients and those with limited mobility, nursed at home or in long-term care facilities. These patients must be reached for VTE screening wherever they live.

The aim of this study was to estimate the prevalence of deep vein thrombosis (DVT) detected by systematic compression ultrasound (CUS) examination in nonacute patients confined to bed or with very reduced mobility, cared for at home or in long-term residential facilities. We also tried to estimate the impact of VTE risk factors on DVT.

## 2. Materials and Methods

The study was conducted in a population of patients nursed at home and in two long-term care facilities in the Vimercate area. Two qualified angiologists examined each patient's proximal veins using portable US machines, and data on risk factors were recorded by a nurse unaware of the results. The study protocol was approved by the local ethics committee, and written informed consent was obtained for each patient.

### 2.1. Patients

The study population included all eligible patients cared for at home with national health service assistance in the Vimercate area between September and December 2007 and all eligible patients resident in the two nursing homes that had expressed willingness to take part, during the winter of 2007-2008. Inclusion criteria were age more than 18 years, inability to attend US screening for DVT in a hospital on account of markedly reduced mobility consisting of total bed rest without bathroom privileges or needing help for restroom, and written consent from the patient or the legal guardian. Patients were excluded if they suffered any acute illness at admission or if they were receiving long-term anticoagulant therapy or prophylaxis. Patients using elastic stockings were eligible.

### 2.2. Data Collection

Data were collected by independent nurses who were given instructions on definitions and data collection techniques. The data included socio-demographic details and predefined risk factors for VTE including any past episodes of VTE, current cancer, chronic respiratory failure, chronic heart failure, neurodegenerative disorders, previous paralytic stroke that remained symptomatic, and reduced mobility, specifying the cause, how long ago it had started (months of immobilization), and the degree of immobility, distinguishing totally bedridden and sedentary patients from those who could still use the bathroom.

The number of hours spent in bed was recorded, and a note was made of whether the patient had edema in one or both legs.

### 2.3. Ultrasound Examination

All the examinations were done by two experienced angiologists using a portable US machine. Venous compression ultrasonography (CUS) was done as described previously [5], using 3- to 7.5-MHz transducers. The common femoral vein in the groin and the popliteal vein extending down to the trifurcation of the calf veins were examined. The only criterion for diagnosing DVT was failure of the vein to collapse completely under compression by the US probe. To minimize the variability due to having two sonographers, about half the patients nursed at home and half the long-term care residents were examined by both angiologists. The decision to give anticoagulant therapy to patients with positive results was left to the treating physician.

### 2.4. Statistical Methods

Logistic regression was used to estimate the impact of VTE risk factors on the probability and subsequent occurrence of DVT. Relative risks were estimated adopting a logarithmic link function. Nonlinear effects were evaluated using restricted cubic spline functions. Putative risk factors were investigated by model selection based on information criteria (AIC). The all possible regression strategy was adopted, considering the best model with a maximum of four risk factors starting from the ten considered [6] because of the low number of events. The stability of the selection procedure was examined by repeating the model selection strategy over 200 bootstrap samples. The model and the variables most selected out of the bootstrap samples were recorded. The discriminant ability of the model was measured by means of the area under the ROC curve by correcting for optimism using bootstrap samples [7]. The probability of DVT for different combinations of risk factors can be estimated from the estimated regression coefficients. 

As an easy-to-use tool to compute the probability of VTE, graphically summarizing the estimated model, a nomogram was obtained starting from the logistic model results. The nomogram is particularly informative on the impact of the different risk factors. The value of each risk factor is translated into a score which is directly connected to the probability of VTE and the most important risk factors are those that give the highest scores.

## 3. Results

Of the 251 patients considered, 221 were eligible for the study. Three were considered ineligible because they refused to give informed consent, 12 were taking anticoagulants, and 15 were still too mobile for admission. Seventy patients were nursed at home and 151 were residents in the nursing homes. Table 1 sets out their main characteristics.

None of the patients were using elastic stockings for prophylaxis.

The CUS examination detected proximal DVT in 40 patients (18%; 95% CI, 13–24%). Three had bilateral thrombosis. There were no cases of symptomatic venous thrombosis or pulmonary embolism.

Table 2 shows the univariate analysis of the putative clinical risk factors. In this (unadjusted) analysis, nonlinear effects were not evidenced by the spline functions for the variables “time of onset of reduced mobility,” “age,” and “hours/day spent in bed.” “Time of onset of reduced mobility,” long-term care versus nursing at home and “causes of reduced mobility” had a significant impact on the risk of DVT. 

The risk of DVT rose by about 1 per 1000 for each month extra in the “time of onset of reduced mobility”. The risk of DVT for patients nursed at home was 24% of that of patients in a long-term care facility. The risk of DVT for patients with causes of reduced mobility other than cognitive impairment was about half the risk for patients with cognitive impairment/dementia (Table 2). 

The model selected through AIC with at most four variables included exactly four variables, namely: Long-term residential care, Previous VTE, Time of onset of reduced mobility, and Causes of reduced mobility. This model was also the one most selected out of 200 bootstrap samples (17.5%). The second most selected model (7.5%) included Long-term residential care, Previous VTE, Bathroom with help, and Causes of reduced mobility. Long-term residential care (97%), Previous VTE (70%), Time of onset of reduced mobility (42%), and Causes of reduced mobility (64%) were the variables most selected in bootstrap samples.  Table 3 shows the estimated relative risks from the logistic model with logarithmic link.

The risk factors had a significant impact. As regards the discriminant ability of the model, the area under the ROC curve was 0.709 with a correction for optimism of about 0.021, resulting in a corrected index of 0.688. The risk factors had slightly more impact in the adjusted model than the unadjusted ones. This was particularly evident for previous VTE. 

**Figure 1:**
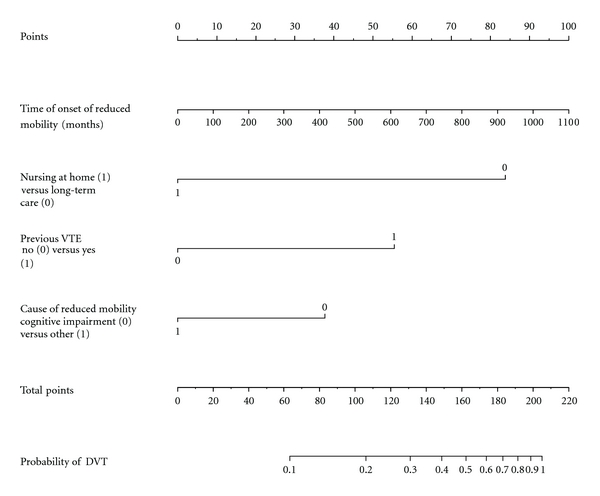
Nomogram to estimate the probability of DVT.

The nomogram in Figure 1 can be used to estimate the probability of proximal DVT. As an example, a patient nursed at home has 0 points, previous VTE has about 55 points, time of onset of reduced mobility 500 (months) has about 45 points, and causes of reduced mobility other than cognitive impairment/dementia have 0 points. These add up to 100 points which corresponds to a probability of about 0.2.

The nomogram also shows that the item with the highest impact among the categorical risk factors is nursing at home versus long-term residential care. Being in a nursing home accumulates 85 points out of a maximum of about 275.

## 4. Discussion

Data on the frequency of VTE among nonacute patients nursed at home or in long-term care residential homes are still scarce. Most reports refer mainly to elderly cases, generally with reduced or no mobility, who cannot easily be screened instrumentally for asymptomatic DVT, unless a sonographer goes to their bedside for the examination, as was done in the present study. However, in a study conducted ten years ago, the incidence of symptomatic VTE based on the Kansas state database for retrospective cohorts of patients nursed at home was 1.30 events per 100 person-years of observation [8]; the mean followup was 233 days and the patients' mean age was 85 years. Substantially, similar findings, also in a historical cohort of patients of about the same average age nursed at home, come from a study in Israel [4] and one from the Mayo Clinic [9]. These figures are far from negligible and suggest that there might have been a much higher rate of asymptomatic VTE in these same patients.

Our estimate of prevalence is the first to date and the 18% of proximal DVT we found is a very high frequency which has never been described before in nonacutely ill patients who in fact, did not have DVT triggering events or so-called exposing risk factors. This elderly study population has an accumulation of predisposing risk factors, though in none of the persons screened VTE was a concern. The percentage we report is similar to that described by Bosson et al. [10] who found a prevalence of more than 15% of systematically detected DVT in patients hospitalized in subacute care facilities, but it is much harder to establish its clinical meaning and the clinical consequences. Nevertheless, it is likely that awareness of the fact that the veins were not compressible in patients like those we studied would induce many treating physicians to consider at least antithrombotic prophylaxis.

Similarly to what has been reported for patients nursed at home [4], our findings suggest that the risk of DVT does not seem to rise just with age or prolonged mobility. In fact one of the most important determinants of this risk appears to be residence in a nursing home rather than living at home. Although we could find no significant interaction (data not shown) between the other variables examined (time of onset of the reduced mobility, previous VTE, and causes of reduced mobility) and being looked after at home or in a long-term care facility, it does seem likely that, in Italy at least, the long-term care residents require more health care and social attention than the patients nursed at home. Possibly beneath these two different profiles lie certain conditions favoring VTE, that we did not detect. It is worth recalling here that Leibson et al. [8], in a population of nursing home residents, found a higher risk of VTE among those requiring more assistance in activities of daily living like grooming, toileting, transferring, positioning in bed, and wheelchair use or those needing physical therapy, rehabilitation, and clinical monitoring. 

Another important point is that cognitive impairment and dementia as a cause of the reduced mobility contributed more to the risk of DVT than other causes such as neurologic paralysis or chronic osteoarticular problems. No easy physiopathological explanation comes to mind, but generally in these patients who are quite different from those with acute illness, the risk factors for VTE seem very different too.

Since the overall DVT risk profile seems so different and cannot easily be assessed by instrumental screening in these patients, we thought that clinicians who manage these cases might find it useful to have a tool like a nomogram that they could consult to identify those at highest risk who could then be kept under closer surveillance, with all the necessary mechanical or pharmacological measures for prevention.

## 5. Study Limitations

One of the main limits of this study is that we could not distinguish recent forms of DVT from older ones where the vessel involved had not been recanalized. Edema and previous VTE did not differ significantly in patients with and without DVT, so these clinical indicators cannot be used to distinguish recent or earlier forms. Therefore, the unexpectedly high prevalence of proximal DVT we observed might simply reflect the earlier development of a thrombotic disorder that had arisen far in advance of the low-mobility status (as a consequence of surgical interventions, hormonal treatment, and so on). The clinical consequences of this are hard to predict, and unfortunately we are unable to provide information on how many patients were treated with prophylactic or therapeutic anticoagulants after the diagnosis was communicated to the treating physician.

Another limitation is that the diagnosis of asymptomatic DVT was based on sonography, not on the standard method, which is venography. Apart from the limited accuracy of portable US devices, sonography has very limited sensitivity in identifying venous thrombosis in asymptomatic post-surgical cases [11], though it appears to be more sensitive and specific in medical patients, as reported by Bressolette et al. [12]. Certainly the pragmatic nature of this study we meant could not have the US results validated by an outside assessor group, and the cases of venous incompressibility were not confirmed by phlebography. However, since CUS is anyway less sensitive than phlebography, our findings are not likely to have overestimated the frequency of DVT.

A further potential limit is that not all the patients examined had been nursed at home or in long-term residential care for the same time. The time of onset of reduced mobility and its severity that we recorded can therefore only be considered a proxy of this information; as we cannot exclude the possibility that this might in fact influence the risk of DVT. The fact that we did not record it may limit the external validity of our prevalence figure. Finally, the model that establishes the most reliable profile of the patient at risk for DVT, and the nomogram we propose, need to be validated in a prospective study on a similar population.

## 6. Conclusions

The risk of proximal DVT in patients nursed at home or in long-term care facilities is a neglected issue, but probably not negligible. Some risk factors such as previous VTE, and especially cognitive impairment as the cause of reduced mobility, or long-term residential care rather than at home, could predispose these subjects to VTE. The prognostic impact of even asymptomatic DVT in nonsurgical patients is important in terms of increased mortality [13], and mortality after VTE is higher for patients nursed at home than for long-term care facility residents [14], so the former should be considered for prophylaxis. However, the risk of pharmacologically-induced bleeding complications has always to be weighed against the possible benefit of preventing a VTE since prophylaxis in this setting will presumably be needed for a long time. Prophylaxis using mechanical means such as compression stockings is not always practical on account of poor compliance, and their efficacy remains to be proved [15]. 

The management of the VTE risk in nonacute patients nursed at home or in long-term care still poses challenging questions, and ad hoc clinical trials are needed to answer them.

## Figures and Tables

**Table:** (a) Continuous variables

Variable	Min–Max	Mean	Median	Q1–Q3
Age (years)	23–102	85.67	88	82–92
Hours/day spent in wheelchair or chair	0–4	8	7.217	6–8
Hours/day spent in bed	10–24	16.78	16	16–18
Time of onset of reduced mobility (months ago)	0–1042	38.28	18	6–36

**Table:** (b) Categorical variables

Variable	No. of patients	%
Cause of limited mobility		
Cognitive impairment/dementia	98	44.3
Neurodegenerative disease/ paralytic stroke	47	21.3
Osteoarticular disease	48	21.7
Other	28	12.7

COPD		
No	190	86.0
Yes	31	14.0

Chronic heart failure		
No	84	38.0
Yes	137	62.0

Cancer		
No	212	95.9
Yes	9	4.1

Cerebrovascular disease		
No	177	80.1
Yes	44	19.9

Previous VTE		
No	196	88.7
Yes	25	11.3

Bathroom with help		
No	128	57.9
Yes	93	42.1

Walking with help		
No	173	78.3
Yes	48	21.7

Leg edema		
No	160	72.4
Yes	61	27.6
Bilateral	55	90.2
Unilateral	6	9.8

**Table 2:** Unadjusted analysis: results of the logistic model with log link to obtain relative risks for each risk factor. For each categorical variable the reference category is indicated by a relative risk of 1 and the corresponding risk of DVT is reported in brackets.

Variable	Relative risk	95% Confidence interval	*P*-value
Hours/day spent in bed	1.031	0.940–1.132	.511
Time of onset of reduced mobility	1.001	1.000–1.002	.025
Age	1.007	0.980–1.035	.622

Cardiovascular disease°			
No	1 (0.190)	0.123–0.296	
Yes versus No	0.920	0.519–1.628	.074

Long-term residential care	1 (0.238)	0.179–0.317	
Nursed at home versus long-term care	0.240	0.089–0.647	.005

Bathroom with help			
No	1(0.203)	0.144–0.286	
Yes versus No	0.741	0.410–1.340	.321

Previous VTE			
No	1(0.173)	0.128–0.235	
Yes versus No	1.384	0.646–2.963	.403

Causes of reduced mobility			
Cognitive impairment/dementia	1(0.255)	0.182–0.358	<.001
Other versus cognitive impairment/dementia	0.478	0.267–0.856	.013

COPD			
No	1 (0.179)	0.132–0.243	
Yes versus No	1.082	0.496–2.361	.844

Leg edema			
No	1 (0.189)	0.137–0.260	
Yes versus No	0.855	0.445–1.642	.638

°Chronic heart failure and previous non-disabling stroke taken together. Previous disabling stroke is considered in the group “causes of reduced motility.”

**Table 3:** Adjusted analysis: results of the logistic model with log link to obtain relative risks for each risk factor.

	Relative risk	95% confidence interval	*P*-value
Time of onset of reduced mobility	1.001	1.000–1.003	.018

Long-term residential care			
Nursed at home versus long-term care	0.257	0.095–0.695	.007

Previous VTE			
Yes versus No	2.454	1.203–5.006	.014

Causes of reduced mobility			
Other versus cognitive impairment/ dementia	0.544	0.297–0.996	.048
